# Variability within *L. albus* and *L. angustifolius* Seeds in Dietary Fiber Components

**DOI:** 10.3390/foods13020299

**Published:** 2024-01-17

**Authors:** Elena González, Ana Isabel Carrapiso, Nuria Canibe, Knud Erik Bach Knudsen

**Affiliations:** 1 School of Agricultural Engineering, Universidad de Extremadura, 06007 Badajoz, Spain; malena@unex.es; 2 Department of Animal and Veterinary Sciences, Aarhus University, Blichers Allé 20, Postboks 50, DK-8830 Tjele, Denmark; nuria.canibe@anivet.au.dk

**Keywords:** lupin seeds, dietary fiber, lupin cultivars, legume composition

## Abstract

Lupin seeds have received increased attention due to their applications in the nutrition of humans and livestock. One of their special features is their high content of dietary fiber, which is influenced by the lupin species. No previous studies have focused on the variability in dietary fiber and its fractions within species so far. The aim of this study was to investigate the variability within *L. albus* and *L. angustifolius* (eight cultivars each) in the dietary fiber composition expressed as low-molecular-weight soluble dietary fiber (LMWSDF), soluble and insoluble non-cellulosic polysaccharides, cellulose, and Klason lignin. Additionally, we analyzed the proximate composition and the composition of amino acids and fatty acids. The results showed noticeable variability within both species not only in the total dietary fiber but also in all its fractions, especially in LMWSDF, cellulose, non-starch polysaccharides, and Klason lignin within *L. angustifolius*. This indicates that the cultivar choice should be based on the application for which it is used. Even though important nutrients, such as the most indispensable amino acids, are not highly variable within *L. albus*, dietary fiber variations can still have a marked influence on the nutritional value because of their influence on the digestibility of other nutrients.

## 1. Introduction

In recent decades, the use of lupin seeds for both human and animal nutrition has received increased attention, with several reviews published recently [1,2,3,4,5,6]. As part of the human diet, recent research revealed health benefits, such as the prevention of diabetes, hypertension, and inflammation [5,7]. In this respect, lupin seeds are rich in dietary fiber (DF) and phytochemicals, the latter with beneficial bioactivity [8]. In addition, their high content of protein and soluble fiber (providing a high-water binding capacity, which favors appetite suppression) and no starch content [9,10] make these seeds a great alternative to obtaining low-sugar, low-energy, and high-fiber food products. Besides appetite control, additional benefits have been reported for lupin fiber, such as antioxidant, prebiotic, and immunostimulatory activities [11]. With respect to animal diets, low alkaloid content lupin cultivars are a suitable alternative to imported soybeans [12,13]. These cultivars, so-called sweet lupins, still include alkaloids, depending on factors such as the species, cultivar, and pedoclimatic conditions (for example, *L. albus*: 3.63–165 mg/100 g; *L. luteus*: 42.6–58.5 mg/kg; *L. angustifolius* 0.36–0.56 mg/kg). Despite their relatively low alkaloid content [4], lupin seeds can have a detrimental effect on animal growth [14]. Some of the drawbacks to lupin-based diets for animal nutrition have been attributed to their high content of DF, which is considered an anti-nutritional factor since it decreases the digestible energy content [15].

Due to the great interest in the lupin DF for obtaining healthier products for humans and its drawbacks for livestock farming, its chemical composition has received increased attention and some factors that may affect it have been researched, such as the influence of species and dehulling [4]. In this respect, a recent study characterized the DF of one cultivar of *L. luteus*, *L. angustifolius*, and *L. albus* [16], whereas several studies have researched the differences between species in the crude fiber [9,17] and the total DF [10,16] content. Until now, no studies have focused on the variability within species of DF and their fractions despite the variability that has been found in their composition, e.g., in their protein and oil content [9]. In this respect, marked variability might be expected, considering the differences reported in the proximate composition between several cultivars [9] and in the non-starch polysaccharides between two cultivars of *L. luteus* and *L. angustifolium*. This variability is worth researching due to its influence on the functional properties of lupin seeds and their nutritional value. These aspects also influence the applications of each cultivar for food products with desired characteristics.

The aim of this investigation is to study the variability in DF components within *L. albus* and *L. angustifolius* seeds. The principal conclusions of this study are that the variability within each species can be noticeable not only in the total DF but also in all the DF fractions, especially within *L. angustifolius.* Therefore, the choice of a cultivar should be based on its features for a specific application, e.g., products rich in fiber aimed at weight loss for humans or weight gain for livestock.

## 2. Materials and Methods

### 2.1. Plant Material and Initial Characterization

Lupin seeds from eight cultivars of white lupin (*Lupinus albus*) grown in France (Ares 96, Lublanc 96, Ares 97, Lublanc 97, DTN-12-96, DTN-20-96, CHD-34-96 and Ludet 96) and eight cultivars of blue lupin (*Lupinus angustifolius*) grown in Poland (Emir 97, Polonez 96) and Germany (E 101, Sonet, Bordako 97, Borweta 97, L1 Rastatt and L2 E 97) were used. The white lupin cultivars were delivered by INRAe (Station d’Amelioration des Plantes Fourrageres, Lusignan, France), the blue lupin cultivars were delivered from Germany by Südwestdeutsche Saatzucht (Rastatt, Germany) and those from Poland were delivered by the Royal Veterinary and Agricultural University (Frederiksberg, Denmark) at amounts of 800–1000 g.

The weight of 1000 whole seeds per cultivar was measured. The cotyledon (endosperm and embryo) and hull percentages were calculated after dehulling by hand and drying. Each parameter was measured once.

### 2.2. Chemical Analyses

The whole seeds were finely ground to pass a 0.5 mm screen and were then used in all the chemical analyses. Two replicates were performed per cultivar for all the chemical analyses.

#### 2.2.1. Proximate Composition

The dry matter (DM) and the ash content were determined after drying at 103 °C for 20 h and after incineration in an oven at 550 °C [18], respectively. The crude protein was determined using the Kjeldahl method [18], a Kjell-Foss 16,200 autoanalyzer, and a general conversion factor of 6.25. The crude fat was extracted using diethyl ether after hydrolysis with HCl [19].

#### 2.2.2. Sugars and Dietary Fiber Analyses

Sugars (fructose, glucose, and sucrose) and raffinose-oligosaccharides (raffinose, stachyose, and verbascose, onward termed low-molecular-weight soluble dietary fiber, LMWSDF) were measured via gas–liquid chromatography, as described by Bach Knudsen and Li [20]. Cellulose, the soluble and insoluble non-cellulosic polysaccharides (S-NCP and I-NCP, respectively), and its constituents were determined as alditol acetates using gas–liquid chromatography for the acid-hydrolyzed neutral sugars and using colorimetric method for uronic acids [21]. The Klason lignin was measured gravimetrically as residue-resistant sulphuric acid hydrolysis, according to Theander and Aman [22].

The total non-cellulosic polysaccharides (NCP) was calculated as follows: NCP = glucose + galactose + xylose + arabinose + rhamnose + mannose + fucose + uronic acids.

Since cellulose in its native form is resistant to hydrolysis with 2 M H_2_SO_4_, the difference between swelling or no swelling with 12 M of H_2_SO_4_ followed by hydrolysis with 2 M H_2_SO_4_ can be used to estimate cellulose. Cellulose was calculated as follows:

Cellulose = NSP_Glucose_ (12 M H_2_SO_4_) − NSP_Glucose_ (2 M H_2_SO_4_)

The total non-starch polysaccharides (NSPs) were calculated as the sum of cellulose, S-NCP, and I-NCP, and the dietary fiber (DF) was calculated as [23]

DF = LMWSDF + total NCP + cellulose + Klason lignin.

#### 2.2.3. Amino Acid and Fatty Acid Analyses

The amino acids were analyzed according to Mason et al. [24]. The fatty acids were analyzed after transesterification following the method described by Engberg et al. [25].

### 2.3. Statistical Analysis

The data were subjected to a one-way analysis of variance to study the differences between the two lupin species and another to study the differences within each species. A principal component analysis was performed to evaluate the multivariate variability of the samples and the relationships among the variables. The Pearson test was applied to check the bivariate relationships between the variables. The SPSS v.27 (SPSS Inc., Chicago, IL, USA) statistical package was used to perform the analyses.

## 3. Results

### 3.1. Lupin Characteristics and Composition

The differences between and within the two species in the lupin characteristics and proximate composition were marked.

With respect to the weight of 1000 seeds and the cotyledon and hull percentages, *L. albus* reached the higher values for the two first variables and the lower for the last one (*p* < 0.001 for all of them) (Table S1). Regarding the variability within species in the weight of 1000 seeds, the coefficient of variation for *L. albus* was higher than *L. angustifolius* (20.2% vs. 10.3%). For the cotyledon and hull percentages, the coefficients of variation for both species were similar (1.1% and 5.3%, respectively, for *L. albus*; 1.2% and 3.9%, respectively, for *L. angustifolius*).

The proximate composition was also greatly affected by both the lupin species (all the variables affected) and the variability within each species (all the variables affected within both species) (Table S2). *L. albus* had a higher content of crude protein, crude fat, and ash and a lower content of DF than *L. angustifolius*. The coefficients of variation for the crude protein, crude fat, and ash content were similar for both species (5.2%, 6.7%, and 7.5% for *L. albus*; 6.4%, 7.4%, and 5.6% for *L. angustifolius*, respectively), whereas, for DF, the coefficient of variation was doubly high for *L. angustifolius* than *L. albus* (6.1% vs. 3.6%, respectively).

### 3.2. Dietary Fiber Composition

Both the differences between and within the two species were significant for most of the dietary fiber fractions, and the variability was larger within *L. angustifolius* than between *L. albus*, as detailed below.

The LMWSDF components were affected by both the species (two out of three compounds and the total LMWSDF) and the variability within each species (two out of three within *L. albus* and all of them within *L. angustifolius*) (Table 1). It should be noted that there was noticeable variability within *L. angustifolius*, with the coefficients of variation in the order of 16.1–28.6% (9.4–25.7% for *L. albus*).

Regarding the S-NCP fraction, significant differences between the species (seven out of eight monomers were affected) and within each species (four out of eight within *L. albus* and five out of eight within *L. angustifolius*) were found (Table 2). With respect to the coefficients of variation, they ranged from 6.2% (uronic acids) to 74.1% (xylose) for *L. albus* and from 7.7 to 121.2% (for uronic acids and glucose) for *L. angustifolius*. The most abundant S-NCP compound, galactose, had a similar coefficient of variation in both species (16.4 and 15.2%, respectively) as it did for S-NCP (11.3 and 13.5%, respectively).

As for I-NCP, significant differences between the species (six out of eight compounds affected) and within them (four out of eight within *L. albus* and *L. angustifolius*) were found (Table 3). The coefficients of variation ranged from 4.9% (xylose) to 53.9% (glucose) for *L. albus* and from 3.5% (uronic acids) to 34.6% (rhamnose) for *L. angustifolius*). The most abundant I-NCP compound, galactose, had a roughly similar coefficient of variation in both species (16.2 and 13.2%, respectively), as it did for the total I-NCP (6.2 and 4.6%).

With respect to the cellulose, total NSP, and Klason lignin content, significant differences between the species and within *L. angustifolius* were found, whereas no differences appeared within *L. albus* (Table 4). The coefficients of variation were lower for *L. albus* than *L. angustifolius* (8.7, 3.4, and 19.6% for the former vs. 10.1, 7.5, and 77.1% for the latter, respectively).

Overall, the differences between and within the two species in all the fiber fractions were noticeable. The differences between the species on the LMWSDF fraction were comparable to the ones within the species, whereas it was much higher for S-NCP and I-NCP. With respect to cellulose, NSP, and Klason lignin, the effect of this species and the variability within *L. angustifolius* was noticeable, whereas within *L. albus*, it was not.

### 3.3. Other Macronutrients and their Relationship to the Dietary Fiber

The differences between and within the two species also affected the amino acid profile as follows: the species had a marked effect (13 out of the 18 amino acids were significantly different), whereas only slight differences were found within *L. albus* (only Arg and Cys were affected) and *L. angustifolius* (8 out of the 18 amino acids) (Table S3). It should be noted that most of the indispensable amino acids were affected by the species (all except His and Lys, with all the affected amino acids except Trp being more abundant in *L. albus*), and half of them were significantly different within *L. angustifolius*. Conversely, only one indispensable amino acid (Cys) was different within *L. albus*. The coefficients of variation for the two species were similar (in the 1.2–7.5 range for *L. albus* and 0.6–7.9 for *L. angustifolius*).

**Figure 1:**
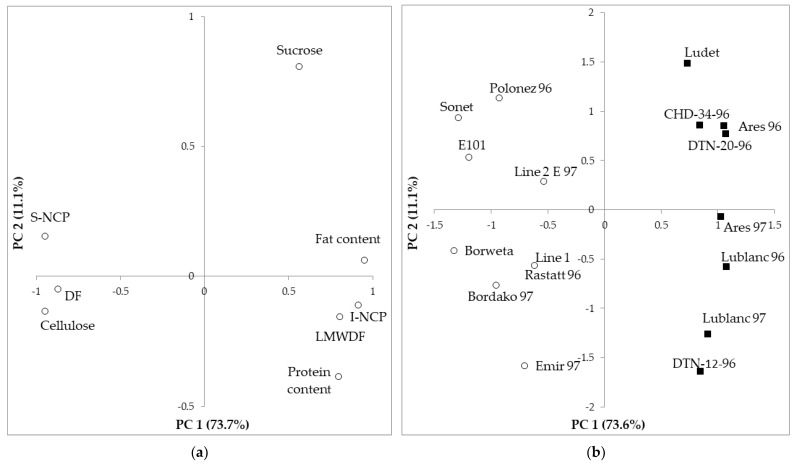
Projection of the variables (**a**) and samples (**b**) onto the space defined by the first two principal components (PC1/PC2). Sample groups: ○ *L. angustifolius*; ■ *L. albus*.

Both the protein content and the amino acid profile were strongly related to the DF content and its fractions. Figure 1a shows that the protein content was positively related to LMWDF and I-NCP (in all the cases with loadings larger than 0.7 in the first principal component, PC 1), whereas it was negatively related to DF, S-NCP, and cellulose (absolute loadings larger than 0.8 in PC 1). The cultivars that appeared in Figure 1b were clearly grouped according to the species, with *L. albus* cultivars reaching high positive scores and the *L. angustifolius* ones reaching highly negative scores in PC 1. In addition, Figure 1b shows that there was noticeable variability within each species. Regarding the protein content, all the correlations involving it were significant (*p* ≤ 0.032 for all of them); the strongest correlations appeared with S-NCP (R: −0.838, *p* < 0.001) and NSP (R: −0.820, *p* < 0.001) and the weakest with LMWSDF (R: 0.536, *p*: 0.032). Six amino acids (Gly, Ile, Leu, Phe, Tyr, and Val) were significantly correlated to the DF content and all its fractions, whereas four (Ala, Arg, His, Lys) were not correlated to any. The strongest correlations involved leucine and I-NCP (R: 0.915, *p* < 0.001) and tyrosine and I-NCP (0.902, *p* < 0.001). The fiber fractions with the strongest correlations to the amino acids were I-NCP and NSP, both with 13 amino acids correlated, and the least correlated were Klason lignin (eight amino acids correlated) and LMWSDF (ten amino acids correlated).

The differences between and within the species on the fatty acid profile were noticeable, with all of them appearing different between and within both species (Table S4). The coefficients of variation for the more abundant fatty acids (above 1% in Table S4) ranged from 5.8 to 20.9% for *L. albus* and from 6.5 to 28.9% for *L. angustifolius*. For the most abundant fatty acid, oleic acid, the coefficients were 5.1% and 12.2%, respectively.

Both the fat content and the fatty acid profile were strongly related to the DF content and its fractions. Figure 1a shows that the fat content was positively related to LMWDF and I-NCP (loadings > 0.7 in PC 1), whereas it was negatively related to DF, S-NCP, and cellulose (loadings > 0.8 in PC 1), similar to the protein content. The fat content was correlated to DF and all its fractions, with the absolute value for R being at least 0.734 and *p* ≤ 0.001; the strongest correlation involved I-NCP (R: 0.940) and cellulose (R: −0.903). Only one fatty acid (20:2n6) was not correlated to DF and any of its fractions, whereas most fatty acids (14 out of 18) were correlated to all of them. The strongest correlation involved NSP and 18:1n9 (R: –0.938, *p* < 0.001) and 18:2n6 (R: 0.927, *p* < 0.001). DF, S-NCP, I-NCP, and NSP were correlated to 17 fatty acids, cellulose, and Klason lignin to 16, and LMWSDF to 14.

The marked differences between and within the two species were found in the mono- and disaccharides, with *p* < 0.001 in all the cases except for the glucose content between species (*p* = 0.675) (Table S5). The coefficients of variation for the two species were roughly similar, ranging from 15.2% to 66.0% for *L. albus* and from 12.2 to 42.3% for *L. angustifolius*. For sucrose (the most abundant compound), the coefficients were similar (15.2% and 14.6%, respectively).

The relationships between these compounds and DF and its components were weaker than the amino acid and fatty acid profiles. Figure 1a shows that the sucrose content followed a similar trend to the protein and fat content, although with a lower score. Glucose was not correlated to any of them, and sucrose was only correlated to cellulose, NSP, and DF (*p* in the 0.011–0.043 range), whereas fructose was correlated to all of them, with *p* in the < 0.001–0.025 range and the strongest correlation involving LMWSDF (R: −0.823, *p* < 0.001) and cellulose (R: 0.775, *p* < 0.001).

To sum up, these results for the major nutrients of lupin seeds show that the differences between and within the two species are noticeable in most of the parameters related to the lupin characteristics and composition, and the protein, fat, and sucrose contents were positively related to LMWDF and I-NCP and negatively to DF, S-NCP, and cellulose, with strong correlations between their components.

## 4. Discussion

### 4.1. Lupin Characteristics and Proximate Composition

The marked difference between these two species in the weight of 1000 seeds, with the values of *L. albus* being twice as high as of *L. angustifolius*, was in line with the differences for the cotyledon and hull percentages, which match previous results [16]. The high variability within both species for the weight of 1000 seeds suggests that there might be considerable differences in the composition of the cultivars and, therefore, in their suitability for human and animal nutrition.

The proximate composition of the whole seeds of *L. albus* and *L. angustifolius* (Table S2) was generally comparable to previous results [9,10,17]. However, it should be noted that slightly different results have also been reported [16], which might be attributed to the differences in the cultivars included in the different studies. This suggests that noticeable species variability may be expected, as found for the proximate composition, where all parameters were affected within both species (Table S2). The higher content of the crude protein and crude fat for *L. albus* than for *L. angustifolius* is in accordance with previous studies [9,17]. It should be noted that, for the protein content, the opposite has also been reported [10,16]. The lower content in DF for *L. albus* is in line with previous studies investigating DF [16], whereas for crude fiber, one study reported a lower content [17], but others reported no differences [9,10]. The lack of consistency for the effect of the species among studies could be related to the cultivars included and to the fact that crude fiber only accounted for a fraction of the total DF [26]. In this respect, the crude fiber includes mainly cellulose and lignin but not LMWSDF and NCP, whereas the dietary fiber includes all of them (LMWSDF includes carbohydrate oligo, with at least a degree of polymerization of two, depending on the country, and polymers [27]).

To sum up, the results confirm that there are noticeable differences between and within the two species in most general parameters.

### 4.2. Dietary Fiber Composition

Until now, few studies have been devoted to researching DF and the factors that influence its content and composition in lupin seeds, especially for the LMWSDF fraction [16]. This is the case despite its great interest in human and animal nutrition. Our results reveal that there is not only variability between but also within species in the total DF content and the DF fractions.

Regarding the LMWSDF fraction (Table 1), the total content was similar to previous results in lupin seeds, with stachyose appearing far more abundant than raffinose and with verbascose in between [16]. The significant differences between the two species in stachyose and verbascose but not in raffinose are also in line with previous work reporting similar differences (55.9%, 40.1%, and only 29.9%, respectively) for the three oligosaccharides between *L. angustifolius* (Sonet *cv.*) and *L. albus* (Feodora *cv.*) [16].

There is no previous information available on whether the cultivar has a significant effect on LMWSDF. The significant differences in the raffinose and verbascose content within *L. albus* and in all the compounds within *L. angustifolius* (where the total LMWSDF content ranged from 39.3 to 79.3 g/kg), together with the relatively large coefficients of variation, suggest that differences related to the cultivar might have a noticeable effect on the seed features and applications. It has been found that oligosaccharides are beneficial for the human diet since they take part in the osmotic regulation in the gastrointestinal tract, reduce the uptake of cholesterol and sugars (which has a positive effect on body weight [15]), and, together with soluble NSP, modify the intestinal passage rate and viscosity [12]. An increase in luminal viscosity may result in lower rates of nutrient absorption and nutritive value [28]. While this is negative in animal nutrition, it is considered beneficial in human nutrition when low-energy diets are desired. Therefore, the repercussions of variations in LMWSDF may be noteworthy, and the choice of the most suitable cultivar within each species and within *L. angustifolius,* in particular for specific use, may be advisable.

The content and composition of S-NCP and I-NCP (Table 2 and Table 3) were generally in agreement with the previous results [16]. The significant differences between the two species in most of the monomers (all except mannose in S-NCP and all except xylose and mannose in I-NCP) generally agree with the previous results. Previous results also reported a higher content in galactose of total NSP when comparing *L. albus* (Hetman *cv.*) and *L. angustifolius* (Saturn and ALS *cv.*) [17] and in S-NCP when comparing *L. angustifolius* (Sonet *cv.*) and *L. albus* (Feodora *cv.*) [16]. However, our results for the galactose content in the I-NCP fraction do not match previous results, where the opposite trend for the insoluble dietary fiber fraction and similar content of galactose were found [16]. The lack of agreement between studies may be related to differences in the analytical methods and in the cultivars included. In this respect, our results showed marked variability within both species (Table 2 and Table 3), with the coefficients of variation for galactose appearing above 13% within both species. This reveals that not only the choice of the species but also the cultivar is critical for the S-NCP and I-NCP content.

No previous investigations on the effect of the cultivar on S-NCP and I-NCP are available. The significant differences found in the S-NCP monomers (four out of eight within *L. albus*; five out of eight within *L. angustifolius*) and the I-NCP monomers (four out of eight within both species), as well as the high coefficients of variation for some monomers, suggest a noticeable source of variation from the cultivar which could potentially have a considerable influence on the nutritive values. However, the varietal effect is much weaker than that of the species, and the latter should, therefore, be carefully considered when S-NCP and I-NCP are crucial for a specific application.

The higher content of cellulose found in *L. angustifolius* (Table 4) does not match the results for the total glucose content in the insoluble DF fraction reported when comparing *L. angustifolius* (Sonet *cv.*) and *L. albus* (Feodora *cv.*) in a previous study [16]. As mentioned before, this lack of agreement might be related to differences in the analytical methodology and to the cultivar differences between studies. With respect to the total NSP and its subfractions (S-NCP, I-NCP, and cellulose), the higher values found in *L. angustifolius* match with previous results comparing *L. albus* (Hetman *cv.*) and *L. angustifolius* (Saturn and ALS *cv.*) [17]. The higher content in Klason lignin in *L. albus* than in *L. angustifolius* (Table 4), however, does not match the previous results reporting the opposite for *L. angustifolius* (Sonet *cv.*) and *L. albus* (Feodora *cv.*), although no statistical analysis was performed [16]. It should be noted, however, that Sonet *cv.* reached a similar content in both studies, which indicates that it is unlikely that this difference would be caused by methodology differences but more likely related to the differences between Feodora *cv.* and the white cultivars included in the current study. This suggests that the cultivar might be a noticeable source of variation for Klason lignin.

The lower values for cellulose and NSP and the higher values for the Klason lignin in *L. albus* than *L. angustifolius* is probably of importance for the digestibility of energy, whereas the differences in NSP and its fractions (S-NCP, I-NCP, and cellulose) can have a marked influence on the functional properties of the seeds and lupin-based products. This is because S-NCP potentially may raise luminal viscosity, thereby reducing the rate of nutrient absorption [29] and NSP in the digestibility of energy [15]. The significant differences in the cellulose, NSP and the Klason lignin content within *L. angustifolius* and the higher variability within *L. angustifolius* than *L. albus* (Table 4) confirms the importance of choosing the cultivar with care for applications, particularly when *L. angustifolius* is involved. Collectively, it is expected that the species and the cultivar might have a noticeable influence on the abovementioned nutritional parameters, as confirmed in several studies in vivo [10,25]. However, further studies should be performed to characterize the cultivars and other potential sources of variation.

### 4.3. Other Macronutrients and Relationship to the Dietary Fiber

The amino acid profile is in line with previous studies, all indicating deficiency in the indispensable amino acid methionine [9,10,17]. The marked effect of this species on the amino acid profile (Table S3) is also in accordance with previous studies [9,16,17]. Differences in indispensable and dispensable amino acids within both species have also been reported [9,10]. The fact that most of the indispensable amino acids are more abundant in *L. albus* confirms a more beneficial amino acid profile than *L. angustifolius,* as also found in a previous study [9]. In addition, the variability within *L. angustifolius* is also important for the nutritive value as six indispensable amino acids were significantly affected (Table S3). These results confirm a noticeably variability not only of the species but also of the cultivar within *L. angustifolius*. Conversely, the choice of a specific lupin cultivar within *L. albus* might have minor nutritional consequences. In addition, our results show strong relationships between the protein content and the amino acid profile with DF and its fractions. Therefore, choosing a cultivar that is high in its DF content may result in a lower protein content and a different amino acid profile with marked influence on the nutritional parameters.

As for the fatty acid profile, there was a marked effect on the species, as also found in other studies [16], with oleic acid and linoleic acids being the most abundant in *L. albus* and *L. angustifolius*, respectively (Table S4). No previous studies have focused on the variability of the fatty acid composition within the lupin species. Our results show that the marked effect within both species (*p* < 0.001 for all the fatty acids) is comparable to the effect of the species itself. The strong relationships between the fat content and the fatty acid profile with DF and its fractions indicate that variations in DF and its fractions may affect the fatty acid and fat content, as mentioned for the amino acids and protein content.

With respect to mono- and disaccharides, the content of sucrose was similar to previous results [16]. However, the higher content of sucrose and total sugars in *L. albus* than in *L. angustifolius* do not match previous results, which reported the opposite for sucrose [9,16] and the nitrogen-free extract [17]. It should be noted that the content of sucrose for the Sonet cultivar (Table S5) was similar to the one previously reported for the same blue lupin cultivar, which was compared with the white Feodora *cv.* [16]. This indicates that the opposite result reported in the study by Keller et al. [16] may be related to the differences between their white lupin cultivar and the ones included in our study, and again suggests that the cultivar might be a noticeable source of variation for sucrose. The significant differences in all the components within both species (*p* < 0.001) and the high coefficients of variation confirm the importance of the cultivar in terms of the sugar content. The strong relationships between some sugars (specifically sucrose) and DF and its fractions suggest that variations in DF may result in variations in the sucrose content, as reported previously for other nutrients.

Taken as a whole, the results confirm that there are noticeable differences between and within these two species for the amino acid and fatty acid profile and the sugar content, including significant differences in most of the indispensable amino acids between species and within *L. angustifolius*. This may have a marked repercussion on the nutritional value of food and feeds based on the lupin seeds.

## 5. Conclusions

In conclusion, our results showed a noticeable varietal source of variation in most DF components within each species, especially within *L. angustifolius*. This variability also appeared in other nutrients, such as the most indispensable amino acids, which were more variable within *L. angustifolius* than within *L. albus*. Our results suggest that variations in DF and its fractions might affect the nutritional parameters directly (because of their own effect) and indirectly (because of their relationships with other component’s content). Therefore, the choice of a cultivar should be based on its features for a specific application, e.g., products rich in fiber aimed at weight loss for humans or weight gain for livestock.

## Figures and Tables

**Table 1:** Low-molecular-weight soluble dietary fiber (LMWSDF) (g/kg dry matter) *.

	Raffinose	Stachyose	Verbascose	Total
Ares 96	6.1 ± 0.5	63.0 ± 5.7	14.1 ± 0.9	83.1 ± 7.1
Ares 97	4.8 ± 0.0	71.8 ± 1.6	7.9 ± 0.3	84.5 ± 1.3
Lublanc 96	5.4 ± 0.1	69.2 ± 7.8	10.6 ± 0.1	85.1 ± 7.6
Lublanc 97	6.5 ± 0.4	72.8 ± 6.7	13.5 ± 0.0	92.8 ± 7.1
CHD-34-96	6.5 ± 0.1	65.8 ± 6.7	8.0 ± 0.1	80.2 ± 6.8
DTN-12-96	5.7 ± 0.5	56.9 ± 1.3	9.6 ± 0.3	72.1 ± 2.1
DTN-20-96	4.4 ± 0.4	57.1 ± 10.7	8.5 ± 0.2	70.0 ± 10.1
Ludet	5.4 ± 0.3	62.8 ± 2.3	7.6 ± 0.1	75.8 ± 1.9
Mean ± SD	5.6 ± 0.8	64.9 ± 6.1	10.0 ± 2.6	80.4 ± 7.5
Coefficient of variation (%)	13.6	9.4	25.7	9.4
*p* (within *L. albus*)	0.002	0.186	<0.001	0.082
Emir 97	5.8 ± 0.1	47.8 ± 2.8	25.7 ± 0.1	79.3 ± 2.8
Polonez 96	6.4 ± 0.1	45.1 ± 1.4	16.1 ± 0.5	67.5 ± 1.0
E 101	4.9 ± 0.1	22.3 ± 0.7	12.1 ± 0.3	39.3 ± 1.1
Sonet	5.3 ± 0.1	27.4 ± 0.2	25.1 ± 0.6	57.7 ± 0.7
Bordako 97	3.5 ± 0.2	26.0 ± 2.1	18.9 ± 0.1	48.3 ± 2.1
Borweta 97	5.2 ± 0.1	29.0 ± 0.9	19.2 ± 0.6	53.3 ± 1.6
L1 Rastatt 96	5.3 ± 0.4	29.9 ± 2.3	27.6 ± 0.6	62.8 ± 3.2
L2 E 97	5.2 ± 0.1	30.0 ± 0.1	20.0 ± 0.1	55.1 ± 0.1
Mean ± SD	5.2 ± 0.8	32.2 ± 9.2	20.6 ± 0.5	57.9 ± 12.2
Coefficient of variation (%)	16.1	28.6	25.5	21.1
*p* (within *L. angustifolius*)	<0.001	<0.001	<0.001	<0.001
*p* (*L. albus* vs. *L. angustifolius*)	0.152	<0.001	<0.001	<0.001

* The results are expressed as the mean ± standard deviation (SD) and significance (*p*) from a one-way ANOVA.

**Table 2:** Soluble non-cellulosic polysaccharides (S-NCP) (g/kg dry matter) *.

	Rhamnose	Fucose	Arabinose	Xylose	Mannose	Galactose	Glucose	Uronic Acids	Total
Ares 96	1.2 ± 0.2	0.8 ± 0.1	20.3 ± 2.6	0.7 ± 0.1	4.0 ± 0.7	72.5 ± 6.2	6.8 ± 1.2	23.4 ± 1.7	129.7 ± 1.6
Ares 97	1.1 ± 0.2	0.6 ± 0.1	17.4 ± 2.2	2.7 ± 0.4	3.5 ± 0.6	59.4 ± 5.0	15.0 ± 2.8	21.4 ± 1.5	121.1 ± 5.3
Lublanc 96	0.9 ± 0.1	0.2 ± 0.0	14.3 ± 1.8	1.3 ± 0.2	3.7 ± 0.6	50.9 ± 4.3	8.6 ± 1.6	21.2 ± 1.5	101.0 ± 6.9
Lublanc 97	1.2 ± 0.2	0.7 ± 0.1	16.2 ± 2.1	2.3 ± 0.3	4.3 ± 0.7	57.3 ± 4.9	7.4 ± 1.4	23.0 ± 1.6	112.3 ± 2.4
CHD-34-96	1.2 ± 0.2	0.6 ± 0.1	21.0 ± 2.7	2.5 ± 0.3	2.9 ± 0.5	76.3 ± 6.5	5.1 ± 0.9	23.8 ± 1.7	133.4 ± 11.4
DTN-12-96	1.3 ± 0.2	0.6 ± 0.1	18.4 ± 2.3	3.4 ± 0.5	4.2 ± 0.7	61.1 ± 5.2	3.3 ± 0.6	20.5 ± 1.4	112.8 ± 7.2
DTN-20-96	1.3 ± 0.2	0.5 ± 0.1	17.9 ± 2.3	0.0 ± 0.0	3.7 ± 0.6	61.7 ± 5.2	3.1 ± 0.6	22.7 ± 1.6	110.9 ± 8.2
Ludet	1.7 ± 0.2	1.0 ± 0.2	22.4 ± 2.9	0.4 ± 0.0	4.0 ± 0.7	82.3 ± 7.0	5.4 ± 1.0	24.5 ± 1.7	141.6 ± 10.3
Mean ± SD	1.2 ± 0.2	0.6 ± 0.2	18.5 ± 2.7	1.6 ± 1.2	3.8 ± 0.4	65.2 ± 10.7	6.8 ± 3.8	22.6 ± 1.4	120.4 ± 13.6
Coefficient of variation (%)	18.3	39.9	14.4	74.1	11.7	16.4	55.5	6.2	11.3
*p* (within *L. albus*)	0.060	0.002	0.108	<0.001	0.526	0.006	0.001	0.289	0.008
Emir 97	2.3 ± 0.4	0.9 ± 0.1	24.7 ± 1.0	0.0 ± 0.0	4.4 ± 0.2	130.1 ± 16.6	12.0 ± 1.2	28.3 ± 4.4	202.5 ± 10.7
Polonez 96	2.7 ± 0.5	1.0 ± 0.1	30.1 ± 1.3	0.6 ± 0.1	3.9 ± 0.3	155.5 ± 19.8	2.6 ± 0.3	30.2 ± 4.7	226.7 ± 25.7
E 101	2.7 ± 0.5	0.9 ± 0.1	30.1 ± 1.3	1.2 ± 0.2	3.3 ± 0.2	129.1 ± 16.4	3.0 ± 0.3	33.3 ± 5.2	203.5 ± 20.0
Sonet	3.1 ± 0.5	1.2 ± 0.2	35.1 ± 1.5	1.7 ± 0.2	4.5 ± 0.3	166.4 ± 21.2	0.9 ± 0.1	35.5 ± 5.5	248.3 ± 14.8
Bordako 97	2.6 ± 0.4	0.8 ± 0.1	27.3 ± 1.2	0.1 ± 0.0	3.1 ± 0.2	129.7 ± 16.5	4.4 ± 0.4	31.8 ± 4.9	199.7 ± 13.9
Borweta 97	2.2 ± 0.4	1.1 ± 0.1	26.6 ± 1.1	0.8 ± 0.1	2.4 ± 0.2	147.6 ± 18.8	2.7 ± 0.3	32.6 ± 5.1	215.9 ± 24.9
L1 Rast	2.4 ± 0.4	0.7 ± 0.1	22.7 ± 1.0	0.4 ± 0.1	4.5 ± 0.3	114.1 ± 14.5	0.0 ± 0.0	31.2 ± 4.8	175.7 ± 18.4
L2 E97	2.0 ± 0.3	0.6 ± 0.1	21.3 ± 0.9	0.0 ± 0.0	2.1 ± 0.1	106.0 ± 13.5	0.0 ± 0.0	28.6 ± 4.5	160.4 ± 8.8
Mean ± SD	2.5 ± 0.3	0.9 ± 0.2	27.2 ± 4.5	0.6 ± 0.7	3.5 ± 1.0	134.8 ± 20.5	3.2 ± 3.9	31.4 ± 2.4	204.1 ± 27.6
Coefficient of variation (%)	13.6	20.9	16.5	102.0	27.1	15.2	121.2	7.7	13.5
*p* (within *L. angustifolius*)	0.371	0.017	<0.001	<0.001	<0.001	0.087	<0.001	0.824	0.023
*p* (*L.albus* vs. *L.angustifolius*)	<0.001	0.001	<0.001	0.003	0.378	<0.001	0.010	<0.001	<0.001

* The results are expressed as the mean ± standard deviation (SD) and significance (*p*) from a one-way ANOVA.

**Table 3:** Insoluble non-cellulosic polysaccharides (I-NCP) (g/kg dry matter) *.

	Rhamnose	Fucose	Arabinose	Xylose	Mannose	Galactose	Glucose	Uronic Acids	Total
Ares 96	1.9 ± 0.2	1.4 ± 0.1	26.0 ± 1.1	31.9 ± 5.4	3.8 ± 0.6	62.2 ± 5.3	8.1 ± 0.6	15.8 ± 1.8	151.0 ± 2.9
Ares 97	2.0 ± 0.2	1.4 ± 0.1	26.7 ± 1.1	32.2 ± 5.5	4.3 ± 0.7	64.3 ± 5.5	2.6 ± 0.2	16.3 ± 1.8	149.7 ± 2.4
Lublanc 96	2.1 ± 0.2	1.8 ± 0.1	27.9 ± 1.2	31.4 ± 5.3	4.5 ± 0.7	73.2 ± 6.2	4.1 ± 0.3	16.2 ± 1.8	161.2 ± 8.8
Lublanc 97	1.8 ± 0.2	1.4 ± 0.1	25.7 ± 1.1	33.5 ± 5.7	5.0 ± 0.8	69.8 ± 5.9	5.5 ± 0.4	17.2 ± 1.9	159.9 ± 14.0
CHD-34-96	1.9 ± 0.2	1.7 ± 0.1	25.7 ± 1.1	35.0 ± 5.9	5.7 ± 0.9	54.7 ± 4.6	12.8 ± 0.9	14.3 ± 1.6	151.7 ± 7.8
DTN-12-96	1.7 ± 0.2	1.4 ± 0.1	26.0 ± 1.1	30.2 ± 5.1	5.0 ± 0.8	52.2 ± 4.4	9.6 ± 0.7	14.6 ± 1.7	140.7 ± 3.1
DTN-20-96	1.7 ± 0.2	1.5 ± 0.1	25.4 ± 1.1	32.5 ± 5.5	5.5 ± 0.9	51.6 ± 4.4	16.8 ± 1.2	14.5 ± 1.6	149.5 ± 0.2
Ludet	1.4 ± 0.1	1.2 ± 0.0	20.5 ± 0.9	34.4 ± 5.8	4.2 ± 0.7	46.1 ± 3.9	11.8 ± 0.8	13.7 ± 1.5	133.2 ± 0.8
Mean ± SD	1.8 ± 0.2	1.5 ± 0.2	25.5 ± 2.2	32.6 ± 1.6	4.7 ± 0.7	59.3 ± 9.6	8.9 ± 4.8	15.3 ± 1.2	149.6 ± 9.2
Coefficient of variation (%)	12.6	13.9	8.5	4.9	14.0	16.2	53.9	7.9	6.2
*p* (within *L. albus*)	0.064	<0.001	0.005	0.987	0.265	0.006	<0.001	0.512	0.041
Emir 97	1.8 ± 0.3	1.9 ± 0.1	21.8 ± 4.0	31.5 ± 2.2	4.5 ± 0.5	42.3 ± 7.8	3.9 ± 0.2	11.2 ± 1.9	118.8 ± 11.9
Polonez 96	0.9 ± 0.1	1.1 ± 0.1	19.5 ± 3.6	31.0 ± 2.2	6.4 ± 0.7	34.2 ± 6.3	6.2 ± 0.3	10.1 ± 1.7	109.3 ± 8.6
E 101	0.8 ± 0.1	1.1 ± 0.1	18.8 ± 3.5	34.8 ± 2.5	9.0 ± 1.0	31.2 ± 5.7	4.3 ± 0.2	10.5 ± 1.8	110.7 ± 7.7
Sonet	0.8 ± 0.1	1.1 ± 0.1	20.6 ± 3.8	29.1 ± 2.1	6.4 ± 0.7	38.5 ± 7.1	6.5 ± 0.3	10.1 ± 1.7	113.2 ± 3.1
Bordako 97	0.9 ± 0.2	1.2 ± 0.1	21.0 ± 3.9	32.5 ± 2.3	3.5 ± 0.4	39.6 ± 7.3	3.5 ± 0.2	10.7 ± 1.8	112.9 ± 0.2
Borweta 97	0.9 ± 0.2	1.1 ± 0.1	21.1 ± 3.9	35.2 ± 2.5	4.3 ± 0.5	47.4 ± 8.7	5.4 ± 0.2	10.7 ± 1.8	126.0 ± 11.4
L1 Rast	0.8 ± 0.1	1.2 ± 0.1	21.1 ± 3.9	32.5 ± 2.3	5.6 ± 0.6	35.0 ± 6.4	8.6 ± 0.4	10.8 ± 1.8	115.6 ± 9.6
L2 E97	0.9 ± 0.2	1.2 ± 0.1	19.8 ± 3.6	28.3 ± 2.0	4.6 ± 0.5	39.2 ± 7.2	8.0 ± 0.3	10.7 ± 1.8	112.8 ± 3.5
Mean ± SD	1.0 ± 0.3	1.2 ± 0.3	20.5 ± 1.0	31.9 ± 2.4	5.6 ± 1.7	38.4 ± 5.0	5.8 ± 1.9	10.6 ± 0.4	114.9 ± 5.3
Coefficient of variation (%)	34.6	20.9	4.9	7.6	31.5	13.2	32.4	3.5	4.6
*p* (within *L. angustifolius*)	0.006	0.001	0.991	0.131	0.001	0.490	<0.001	0.998	0.565
*p* (*L. albus* vs. *L. angustifolius*)	<0.001	0.014	<0.001	0.554	0.106	<0.001	0.019	<0.001	<0.001

* The results are expressed as the mean ± standard deviation (SD) and significance (*p*) from a one-way ANOVA.

**Table 4:** Cellulose, non-starch polysaccharides (NSP) and Klason lignin (g/kg dry matter) *.

	Cellulose	NSP	Klason Lignin
Ares 96	78.5 ± 14.4	359.2 ± 13.1	11.4 ± 2.1
Ares 97	92.3 ± 17.0	363.2 ± 14.0	9.2 ± 1.7
Lublanc 96	96.0 ± 17.6	358.2 ± 15.8	12.5 ± 2.3
Lublanc 97	106.9 ± 19.6	379.1 ± 8.0	9.8 ± 1.8
CHD-34-96	98.3 ± 18.1	383.4 ± 14.5	8.5 ± 1.6
DTN-12-96	97.0 ± 17.8	350.5 ± 7.5	13.5 ± 2.5
DTN-20-96	88.9 ± 16.3	349.2 ± 8.0	7.9 ± 1.5
Ludet	92.3 ± 17.0	367.2 ± 5.9	9.1 ± 1.7
Mean ± SD	93.8 ± 8.2	363.8 ± 12.4	10.2 ± 2.0
Coefficient of variation (%)	8.7	3.4	19.6
*p* (within *L. albus*)	0.847	0.128	0.146
Emir 97	126.5 ± 7.2	447.9 ± 5.9	9.0 ± 1.1
Polonez 96	129.5 ± 7.3	465.5 ± 9.8	0.5 ± 0.1
E 101	161.3 ± 9.1	475.5 ± 3.1	2.0 ± 0.3
Sonet	139.1 ± 7.9	500.6 ± 10.1	3.1 ± 0.4
Bordako 97	143.3 ± 8.1	455.9 ± 6.0	4.0 ± 0.5
Borweta 97	164.0 ± 9.3	506.0 ± 4.2	3.2 ± 0.4
L1 Rast	140.7 ± 8.0	432.0 ± 0.8	4.9 ± 0.6
L2 E97	129.3 ± 7.3	402.4 ± 19.6	1.2 ± 0.2
Mean ± SD	141.7 ± 14.3	460.8 ± 34.4	3.5 ± 2.7
Coefficient of variation (%)	10.1	7.5	77.1
*p* (within *L. angustifolius*)	0.009	<0.001	<0.001
*p* (*L. albus* vs. *L. angustifolius*)	<0.001	<0.001	<0.001

* The results are expressed as the mean ± standard deviation (SD) and significance (*p*) from a one-way ANOVA.

## Data Availability

The original contributions presented in the study are included in the article/Supplementary Material, further inquiries can be directed to the corresponding authors.

## Supplementary Materials

The following supporting information can be downloaded at: https://www.mdpi.com/article/10.3390/foods13020299/s1, Table S1: Weight of 1000 seeds, and cotyledon and hull percentages of the lupin seeds *; Table S2: Proximate composition (g/kg dry matter) of the lupin seeds *; Table S3: Amino acids (g/16 g N) of the lupin seeds *; Table S4: Fatty acid profile (%) of the lupin seeds *; Table S5: Mono and disaccharides (g/kg dry matter) of the lupin seeds *; Table S6: Pearson correlation coefficients (significance) between the dietary fiber fractions and other lupin components *.
