# Supplementary material for: Variability within L. albus and L. angustifolius Seeds in Dietary Fiber Components
Source: Foods. 2024 Jan 17;13(2):299. doi: 10.3390/foods13020299 (PMC10814947; doi:10.3390/foods13020299)
Supplement: Supplementary file 1 [file foods-13-00299-s001.zip › foods-2811626-supplementary.pdf]

Table S1. Weight of 1000 seeds, and cotyledon and hull percentages of the lupin seeds*.

	Weight (g) of 1000 seeds	Cotyledon (%)	Hull (%)
Ares 96	433.9	84.3	15.7
Ares 97	294.4	83.2	17.1
Lublanc 96	471.8	84.7	15.3
Lublanc 97	326.6	82.8	17.2
CHD-34-96	262.2	81.9	18.1
DTN-12-96	337.0	82.8	17.2
DTN-20-96	321.5	82.9	17.2
Ludet	398.9	82.8	17.2
Mean ± SD	355.8 ± 72.0	83.1 ± 0.9	16.9 ± 0.9
Emir 97	144.2	76.6	23.4
Polonez 96	149.0	77.5	22.5
E101	160.3	76.1	23.9
Sonet	145.1	78.2	21.8
Bordako 97	179.4	77.0	23.0
Borweta 97	163.2	75.5	24.5
Line 1 Rastatt 96	141.4	76.0	24.0
Line 2 E 97	128.7	76.8	23.2
Mean ± SD	151.4 ± 15.6	76.7 ± 0.9	23.3 ± 0.9
p (*L. albus* vs *L. angustifolius*)	< 0.001	< 0.001	< 0.001

*Only one measurement per cultivar was performed.

Table S2. Proximate composition (g/kg dry matter) of the lupin seeds*.

	Crude protein	Crude fat	Ash	Dietary fiber
Ares 96	350.7^{cd}	129.1^{b}	46.0^{a}	453.6^{fg}
Ares 97	382.9^{b}	117.8^{c}	42.5^{c}	456.9^{efg}
Lublanc 96	355.3^{cd}	133.9^{a}	44.5^{b}	455.8^{fg}
Lublanc 97	375.9^{b}	117.6^{c}	42.3^{c}	481.7^{de}
CHD-34-96	365.1^{bc}	112.0^{d}	38.5^{ef}	472.1 ^{ef}
DTN-12-96	411.2^{a}	110.3^{d}	44.5^{b}	436.1^{gh}
DTN-20-96	380.7^{b}	111.2^{d}	41.0^{d}	427.1^{h}
Ludet	351.0^{cd}	119.7^{c}	36.0^{i}	452.0^{fg}
Mean ± SD	371.6 ± 19.3	119.0 ± 8.0	41.9 ± 3.1	454.4 ± 16.4
p (within *L. albus*)	<0.001	<0.001	<0.001	0.028
Emir 97	337.5^{de}	70.3^{e}	37.5^{gh}	536.2^{b}
Polonez 96	301.9^{f}	68.4^{ef}	37.5^{gh}	533.5^{b}
E 101	326.5^{e}	61.1^{hi}	37.0^{h}	516.8^{bc}
Sonet	296.7^{f}	64.3^{gh}	42.5^{c}	561.4^{a}
Bordako 97	353.3^{cd}	57.8^{i}	39.0^{e}	508.2^{c}
Borweta 97	298.6^{f}	71.9^{e}	42.5 ^{c}	562.4^{a}
L1 Rastatt	353.6^{cd}	59.7^{i}	37.0^{h}	499.7 ^{cd}
L2 E97	320.5^{e}	65.4^{fg}	38.0^{fg}	458.8 ^{efg}
Mean ± SD	323.6 ± 20.6	64.9 ± 4.8	38.9 ± 2.2	522.1 ± 32.1
p (within *L. angustifolius*)	0.001	<0.001	<0.001	<0.001
p (*L. albus* vs *L. angustifolius*)	<0.001	<0.001	0.004	<0.001

*The results are expressed as mean ± standard deviation (SD) and significance (*p*) from a one-way ANOVA. Different letters in the same column indicate significant differences (p <0.05).

Table S3. Amino acids (g/16 g N) of the lupin seeds*.

	His**	Ile**	Leu**	Lys**	Met**	Cys**	Phe**	Tyr**	Thr**	Trp**	Val**	Ala	Arg	Asp	Glu	Gly	Pro	Ser
Ares 96	2.4^{bc}	4.7^{abc}	7.3^{abc}	4.8^{abc}	0.7^{ab}	1.7^{b}	4.1^{ab}	4.7^{ab}	3.7^{a}	0.8^{ef}	4.5^{ab}	3.4^{ab}	9.6^{f}	10.3^{ab}	19.3^{cde}	4.0^{ab}	4.3^{a}	5.5^{a}
Ares 97	2.4^{bc}	4.6^{abc}	7.2^{abcde}	4.7^{abc}	0.7^{ab}	1.5^{cd}	4.1^{ab}	4.7^{ab}	3.6^{abcd}	0.7^{f}	4.3^{bcd}	3.4^{ab}	10.7^{bc}	10.3^{ab}	19.5^{bcde}	4.0^{ab}	4.3^{a}	5.4^{ab}
Lublanc 96	2.5^{bc}	4.6^{abc}	7.5^{a}	4.8 ^{ab}	0.7^{a}	1.9^{a}	4.0^{abcd}	4.6^{b}	3.7^{ab}	0.8^{ef}	4.2^{bcdef}	3.3^{abc}	9.6^{f}	10.4^{ab}	19.5^{bcde}	4.0^{ab}	4.1^{ab}	5.5^{a}
Lublanc 97	2.5^{b}	4.6^{abc}	7.3^{ab}	4.8^{abc}	0.6^{bcd}	1.7^{b}	4.0^{abcde}	4.7^{ab}	3.7^{abc}	0.7^{f}	4.5^{ab}	3.4^{ab}	10.4^{cd}	10.4^{ab}	19.7^{abcde}	4.0^{ab}	4.3^{ab}	5.4^{ab}
CHD-34-96	2.4^{bc}	4.8^{a}	7.4^{ab}	4.^{9a}	0.6^{bcd}	1.5^{cd}	4.2^{ab}	4.9^{a}	3.6^{abcd}	0.8^{ef}	4.3^{abc}	3.4^{ab}	9.9^{ef}	10.6^{a}	19.2^{cde}	4.0^{ab}	4.3^{a}	5.5^{a}
DTN-12-96	2.4^{bc}	4.5^{bcd}	7.1^{abcdef}	4.6^{bc}	0.6^{bcd}	1.6^{bc}	3.9^{bcde}	4.5^{b}	3.5^{bcde}	0.7^{f}	4.1^{cdefg}	3.1^{c}	11.0^{b}	10.1^{ab}	19.0^{de}	3.9 ^{b}	4.1^{ab}	5.2^{abc}
DTN-20-96	2.4^{bc}	4.7^{ab}	7.3^{abc}	4.8^{abc}	0.6^{bcd}	1.7^{b}	4.1^{ab}	4.7^{ab}	3.6^{abcd}	0.8^{def}	4.4^{ab}	3.3^{abc}	10.3^{cde}	10.5^{a}	19.4^{bcde}	3.9^{ab}	4.3^{a}	5.4^{ab}
Ludet	2.4^{bc}	4.8^{a}	7.3^{abcd}	4.9^{a}	0.7^{ab}	1.5^{def}	4.2^{a}	4.8^{a}	3.7^{abc}	0.7^{f}	4.6^{a}	3.4^{ab}	9.6^{f}	10.6^{a}	18.8^{e}	4.0^{ab}	4.4^{a}	5.4^{ab}
Mean ± SD	2.4 ± 0.0	4.7 ± 0.1	7.3 ± 0.1	4.8 ± 0.1	0.7 ± 0.1	1.6 ± 0.1	4.1 ± 0.1	4.7 ± 0.1	3.6 ± 0.1	0.8 ± 0.1	4.4 ± 0.2	3.4 ± 0.1	10.1 ± 0.5	10.4 ± 0.2	19.3 ± 0.3	4.0 ± 0.0	4.3 ± 0.1	5.4 ± 0.1
p (within *L. albus*)	0.762	0.164	0.872	0.351	0.124	<0.001	0.363	0.142	0.295	0.312	0.120	0.092	0.001	0.728	0.412	0.939	0.473	0.763
Emir 97	2.8^{a}	4.1^{f}	6.5^{g}	4.7^{abc}	0.6^{cde}	1.4^{ef}	4.0^{abc}	3.6^{c}	3.4^{e}	0.9^{a}	4.2^{bcde}	3.4^{ab}	9.8^{ef}	9.2^{c}	19.3 ^{bcde}	4.1^{ab}	4.2^{ab}	4.6^{d}
Polonez 96	2.3^{bc}	4.3 ^{def}	6.7 ^{defg}	4.8^{ab}	0.6^{bcd}	1.5^{cde}	3.8^{defg}	3.5^{c}	3.5^{abcde}	0.8^{bcd}	4.0^{defg}	3.4^{ab}	9.9^{def}	10.1^{ab}	20.1^{abcd}	4.1^{ab}	4.2^{ab}	5.2^{abc}
E 101	2.4^{bc}	4.4^{cde}	6.7^{cdefg}	4.7^{abc}	0.6^{fg}	1.4^{f}	3.8^{cdef}	3.7^{c}	3.4^{cde}	0.8^{def}	3.9^{fg}	3.3^{abc}	10.7^{bc}	10.2^{ab}	20.5^{ab}	4.1^{ab}	4.2^{ab}	5.1^{abc}
Sonet	2.3^{bc}	4.3 ^{def}	6.6^{fg}	4.8^{abc}	0.6^{bcd}	1.4^{f}	3.7^{fg}	3.6^{c}	3.5^{abcde}	0.8^{cde}	4.0^{defg}	3.4^{a}	9.6^{f}	10.1^{ab}	19.2^{cde}	4.1^{ab}	4.1^{ab}	5.0^{bc}
Bordako 97	2.3^{bc}	4.3^{def}	6.7^{defg}	4.5^{c}	0.5^{g}	1.4^{f}	3.7^{efg}	3.6^{c}	3.4^{de}	0.8^{ef}	3.8^{g}	3.2^{bc}	10.8^{bc}	10.5^{a}	20.8^{a}	4.1^{ab}	4.1^{ab}	5.2^{abc}
Borweta 97	2.3^{bc}	4.2^{ef}	6.8^{bcdefg}	4.9^{ab}	0.6^{de}	1.6^{bc}	3.7^{fg}	3.7^{c}	3.5^{abcde}	0.9^{bc}	3.9^{efg}	3.4^{ab}	10.3^{cde}	10.0^{ab}	20.1^{abcd}	4.1^{ab}	4.0^{b}	5.3^{abc}
L1 rast	2.7^{a}	4.0^{f}	6.6^{efg}	4.7^{abc}	0.6^{ef}	1.6^{bc}	3.5^{g}	3.3^{d}	3.4^{e}	0.8^{cde}	3.9^{defg}	3.4^{ab}	11.5^{a}	9.6^{bc}	20.4^{abc}	4.1^{ab}	4.3^{a}	5.0^{cd}
L2 E97	2.5^{b}	4.1^{f}	6.8^{bcdefg}	4.7^{abc}	0.7^{abc}	1.7^{b}	3.6^{fg}	3.4^{cd}	3.5^{abcde}	0.9^{ab}	4.0^{defg}	3.4^{ab}	9.9^{def}	9.8^{abc}	19.8^{abcde}	4.2^{a}	4.4^{a}	5.0^{cd}
Mean ± SD	2.5 ± 0.2	4.2 ± 0.1	6.7 ± 0.1	4.7 ± 0.1	0.6 ± 0.1	1.5 ± 0.1	3.7 ± 0.1	3.6 ± 0.1	3.5 ± 0.1	0.8 ± 0.1	3.9 ± 0.1	3.4 ± 0.1	10.3 ± 0.6	9.9 ± 0.4	20.0 ± 0.6	4.1 ± 0.0	4.2 ± 0.1	5.1 ± 0.2
p (within *L. angustifolius*)	<0.001	0.195	0.850	0.131	0.001	0.001	0.009	0.034	0.566	0.002	0.125	0.447	0.001	0.113	0.237	0.991	0.073	0.04
p (*L. albus* vs *L. angustifolius*)	0.528	<0.001	<0.001	0.124	<0.001	0.003	<0.001	<0.001	<0.001	<0.001	<0.001	0.260	0.365	0.003	0.001	<0.001	0.165	<0.001

* The results are expressed as mean and mean ± standard deviation (SD) and significance (*p*) from a one-way ANOVA. Different letters in the same column indicate significant differences (p <0.05).

** Essential amino acids.

Table S4. Fatty acid profile (%) of the lupin seeds *.

	12:0	14:0	15:0	16:0	17:0	18:0	20:0	22:0	24:0	16:1	18:1n9	20:1n9	22:1n9	24:1n	18:2n6	18:3n3	20:2n6	20:3n6	Sat	Mono	Pol
Ares 96	0.0^{f}	0.1^{e}	0.0^{f}	7.6^{fg}	0.0^{e}	2.5^{h}	1.2^{b}	3.2^{c}	0.0^{g}	0.2^{c}	57.9^{a}	4.8^{a}	1.2^{b}	0.0^{c}	14.4^{j}	6.9^{d}	0.0^{d}	0.0^{f}	14.6^{hi}	64.1^{a}	21.3^{j}
Ares 97	0.0^{f}	0.2^{e}	0.0^{f}	7.2^{gh}	0.0^{e}	2.8^{g}	1.5^{a}	3.9^{a}	0.0^{g}	0.3^{b}	56.0^{ab}	3.6^{c}	1.2^{b}	0.0^{c}	12.9^{k}	8.5^{ab}	1.9^{a}	0.0^{f}	15.6^{g}	61.1^{b}	23.3^{i}
Lublanc 96	0.0^{f}	0.2^{e}	0.0^{f}	7.6^{fg}	0.0^{e}	2.4^{h}	1.2^{b}	3.3^{c}	0.0^{g}	0.3^{b}	54.4^{b}	4.1^{b}	1.5^{a}	0.5^{b}	16.5^{i}	8.0^{c}	0.0^{d}	0.0^{f}	14.7^{hi}	60.8^{b}	24.5^{i}
Lublanc 97	0.0^{f}	0.2^{e}	0.0^{f}	7.9^{ef}	0.0^{e}	2.3^{h}	1.2^{b}	3.5^{b}	0.0^{g}	0.4^{a}	56.4^{ab}	3.3^{d}	0.9^{d}	0.0^{c}	15.6^{ij}	8.4^{b}	0.0^{d}	0.0^{f}	15.1^{gh}	61.0^{b}	24.0^{i}
CHD-34-96	0.0^{f}	0.1^{e}	0.0^{f}	7.2^{gh}	0.0^{e}	1.6^{j}	0.8^{f}	2.6^{e}	0.0^{g}	0.3^{b}	49.7^{c}	3.3^{d}	0.9^{d}	0.5^{b}	23.9^{f}	8.7^{a}	0.3^{c}	0.0^{f}	12.3^{j}	54.7^{d}	32.9^{e}
DTN-12-96	0.0^{f}	0.1^{e}	0.0^{f}	8.1^{ef}	0.0^{e}	1.9^{i}	0.9^{d}	3.2^{c}	0.0^{g}	0.4^{a}	51.7^{c}	4.1^{b}	1.5^{a}	0.6^{a}	18.7^{h}	8.4^{b}	0.4^{b}	0.0^{f}	14.2^{i}	58.3^{c}	27.5^{h}
DTN-20-96	0.0^{f}	0.2^{e}	0.0^{f}	8.2^{e}	0.0^{e}	2.0^{i}	0.9^{d}	2.9^{d}	0.0^{g}	0.4^{a}	51.6^{c}	3.0^{e}	0.8^{e}	0.6^{a}	21.4^{g}	8.0^{c}	0.0^{d}	0.0^{f}	14.2^{i}	56.4^{cd}	29.4^{g}
Ludet	0.0^{f}	0.1^{e}	0.0^{f}	6.9^{h}	0.0^{e}	1.8^{ij}	0.9^{d}	2.6^{e}	0.0^{g}	0.3^{b}	51.5^{c}	3.7^{c}	1.0^{c}	0.0^{c}	22.3^{g}	8.6^{ab}	0.3^{c}	0.0^{f}	12.3^{j}	56.5^{cd}	31.2^{f}
Mean ± SD	0.0 ± 0.0	0.2 ± 0.1	0.0 ± 0.0	7.6 ± 0.4	0.0 ± 0.0	2.2 ± 0.4	1.1 ± 0.2	3.2 ± 0.4	0.0 ± 0.0	0.3 ± 0.1	53.7 ± 2.7	3.7 ± 0.5	1.1 ± 0.3	0.3 ± 0.3	18.2 ± 3.7	8.2 ± 0.5	0.4 ± 0.6	0.0 ± 0.0	14.1 ± 1.1	59.1 ± 2.9	26.8 ± 3.9
p (within *L. albus*)	-	<0.001	-	<0.001	-	<0.001	<0.001	<0.001	-	<0.001	<0.001	<0.001	<0.001	<0.001	<0.001	<0.001	<0.001	-	<0.001	<0.001	<0.001
Emir 97	0.1^{b}	0.3^{a}	0.1^{c}	12.1^{b}	0.0^{e}	5.6^{d}	0.6^{h}	1.3^{j}	0.0^{g}	0.0^{h}	34.9^{f}	0.2^{g}	0.0^{f}	0.0^{c}	40.0^{c}	5.3^{gh}	0.0^{d}	0.0^{f}	20.0^{ef}	35.1^{g}	44.9^{c}
Polonez 96	0.1^{a}	0.2^{d}	0.1^{e}	11.2^{de}	0.1^{a}	7.8^{a}	1.0^{c}	2.0^{f}	0.4^{b}	0.1^{d}	38.0^{e}	0.3^{fg}	0.0^{f}	0.0^{c}	34.7^{d}	4.1^{i}	0.0^{d}	0.1^{de}	22.8^{a}	38.4^{f}	38.8^{d}
E 101	0.0^{f}	0.2^{bc}	0.1^{d}	11.9^{bc}	0.1^{a}	7.2^{b}	0.9^{de}	1.9^{g}	0.4^{d}	0.1^{fg}	32.8^{fg}	0.2^{fg}	0.0^{f}	0.0^{c}	38.7^{c}	5.5^{g}	0.0^{d}	0.1^{b}	22.6^{a}	33.1^{gh}	44.3^{c}
Sonet	0.1^{d}	0.3^{b}	0.1^{a}	11.7^{bc}	0.1^{a}	4.7^{e}	0.7^{g}	1.4^{i}	0.3^{e}	0.1^{de}	31.9^{g}	0.2^{fg}	0.0^{f}	0.0^{c}	41.8^{b}	6.6^{e}	0.0^{d}	0.1^{d}	19.3^{f}	32.2^{h}	48.5^{b}
Bordako 97	0.1^{c}	0.2^{c}	0.1^{b}	13.1^{a}	0.1^{c}	4.8^{e}	0.3^{i}	1.7^{h}	0.4^{c}	0.1^{de}	34.5^{f}	0.3^{f}	0.0^{f}	0.0^{c}	38.3^{c}	6.0^{f}	0.0^{d}	0.1^{a}	20.7^{cd}	34.9^{g}	44.4^{c}
Borweta 97	0.1^{e}	0.2^{d}	0.1^{c}	13.0^{a}	0.1^{d}	3.9^{f}	0.6^{h}	1.8^{g}	0.4^{a}	0.1^{g}	29.3^{h}	0.3^{f}	0.0^{f}	0.0^{c}	44.1^{a}	6.0^{f}	0.0^{d}	0.1^{e}	20.2^{de}	29.7^{i}	50.1^{a}
L1 rast	0.0^{f}	0.2^{e}	0.1^{e}	11.4^{cd}	0.1^{ab}	7.3^{b}	0.8^{f}	1.5^{i}	0.3^{f}	0.1^{ef}	44.1^{d}	0.2^{fg}	0.0^{f}	0.0^{c}	28.4^{e}	5.6^{g}	0.0^{d}	0.1^{c}	21.7^{b}	44.4^{e}	34.0^{e}
L2 E97	0.0^{f}	0.2^{e}	0.1^{c}	10.7^{e}	0.1^{bc}	7.0^{c}	0.9^{e}	2.0^{f}	0.4^{d}	0.1^{d}	39.2^{e}	0.2^{fg}	0.0^{f}	0.0^{c}	34.0^{d}	5.2^{h}	0.0^{d}	0.1^{b}	21.3^{bc}	39.5^{f}	39.3^{d}
Mean ± SD	0.1 ± 0.0	0.2 ± 0.0	0.1 ± 0.0	11.9 ± 0.8	0.1 ± 0.0	6.0 ± 1.4	0.7 ± 0.2	1.7 ± 0.3	0.3 ± 0.1	0.1 ± 0.0	35.6 ± 4.4	0.2 ± 0.0	0.0 ± 0.0	0.0 ± 0.0	37.5 ± 4.7	5.5 ± 0.7	0.0 ± 0.0	0.1 ± 0.0	21.1 ± 1.2	35.9 ± 4.4	43.0 ± 5.0
p (within *L. angustifolius*)	<0.001	<0.001	<0.001	<0.001	<0.001	<0.001	<0.001	<0.001	<0.001	<0.001	<0.001	<0.001	-	-	<0.001	<0.001	-	<0.001	<0.001	<0.001	<0.001
p (*L. albus* vs *L. angustifolius*)	<0.001	<0.001	<0.001	<0.001	<0.001	<0.001	<0.001	<0.001	<0.001	<0.001	<0.001	<0.001	<0.001	<0.001	<0.001	<0.001	0.03	<0.001	<0.001	<0.001	<0.001

* The results are expressed as mean and mean ± standard deviation (SD) and significance (*p*) from a one-way ANOVA. Different letters in the same column indicate significant differences ($p < 0.05$). Sat: saturated; Mono: monounsaturated; Pol: polyunsaturated.

Table S5. Mono and disaccharides (g/kg dry matter) of the lupin seeds *.

	Fructose	Glucose	Sucrose	Total
Ares 96	4.4^{e}	3.4^{ab}	355.0^{b}	362.8^{b}
Ares 97	4.1^{e}	2.4^{def}	334.5^{c}	341.0^{c}
Lublanc 96	2.9^{g}	0.0^{g}	290.5^{ef}	293.4^{ef}
Lublanc 97	3.2^{fg}	0.0^{g}	276.0^{fg}	279.2^{fg}
CHD-34-96	3.6^{f}	2.4^{ef}	374.5^{a}	380.4^{a}
DTN-12-96	4.4^{e}	3.0^{bc}	254.5^{h}	261.9^{hi}
DTN-20-96	5.5^{cd}	3.6^{a}	372.5^{a}	381.5^{a}
Ludet	2.8^{g}	2.2^{ef}	384.0^{a}	389.0^{a}
Mean ± SD	3.8 ± 0.9	2.1 ± 1.4	330.2 ± 50.0	336.1 ± 50.9
p (within *L. albus*)	<0.001	<0.001	<0.001	<0.001
Emir 97	5.4^{cd}	2.6^{cdef}	200.0^{j}	207.9^{k}
Polonez 96	5.7^{c}	2.5^{cdef}	317.0^{d}	325.2^{d}
E 101	6.7^{b}	0.0^{g}	297.0^{e}	303.7^{e}
Sonet	5.4^{cd}	3.0^{bcd}	297.0^{e}	305.4^{e}
Bordako 97	5.7^{c}	2.6^{cdef}	243.0^{hi}	251.3^{ij}
Borweta 97	7.2^{a}	2.7^{cde}	234.5^{i}	244.4^{j}
Line 1 Rastatt 96	5.2^{d}	2.1^{f}	269.5^{g}	276.8^{gh}
Line 2 E 97	6.5^{b}	3.0^{bcd}	283.5^{efg}	293.0^{ef}
Mean ± SD	6.0 ± 0.7	2.3 ± 1.0	267.7 ± 39.1	275.9 ± 38.9
p (within *L. angustifolius*)	<0.001	<0.001	<0.001	<0.001
p (*L. albus* vs *L. angustifolius*)	<0.001	0.675	<0.001	<0.001

* The results are expressed as mean and mean ± standard deviation (SD) and significance (*p*) from a one-way ANOVA. Different letters in the same column indicate significant differences (*p* <0.05).

Table S6. Pearson correlation coefficients (significance) between the dietary fiber fractions and other lupin components *.

	LMWSDF	S-NCP	I-NCP	Cellulose	NSP	Klason lignin	DF
S-NCP	-0.683 (0.004)						
I-NCP	0.805 (<0.001)	-0.872 (<0.001)					
Cellulose	-0.816 (<0.001)	0.829 (<0.001)	-0.800 (<0.001)				
NSP	-0.704 (0.002)	0.961 (<0.001)	-0.792 (<0.001)	0.922 (<0.001)			
Klason lignin	0.782 (<0.001)	-0.794 (<0.001)	0.823 (<0.001)	-0.795 (<0.001)	-0.783 (<0.001)		
DF	-0.480 (0.060)	0.916 (<0.001)	-0.655 (0.006)	0.820 (<0.001)	0.960 (<0.001)	-0.636 (0.008)	
Crude protein	0.536 (0.032)	-0.838 (<0.001)	0.672 (0.004)	-0.685 (0.003)	-0.820 (<0.001)	0.778 (<0.001)	-0.789 (<0.001)
Histidine	0.306 (0.249)	-0.070 (0.797)	-0.038 (0.888)	-0.032 (0.907)	-0.087 (0.750)	0.209 (0.437)	0.013 (0.963)
Isoleucine	0.541 (0.037)	-0.671 (0.006)	0.789 (<0.001)	-0.766 (0.001)	-0.683 (0.005)	0.593 (0.020)	-0.611 (0.016)
Leucine	0.661 (0.005)	-0.875 (<0.001)	0.915 (<0.001)	-0.818 (<0.001)	-0.834 (<0.001)	0.683 (0.004)	-0.771 (<0.001)
Lysine	0.318 (0.230)	-0.139 (0.609)	0.359 (0.173)	-0.249 (0.353)	-0.117 (0.667)	0.057 (0.835)	-0.032 (0.905)
Methionine	0.717 (0.002)	-0.533 (0.033)	0.601 (0.014)	-0.701 (0.003)	-0.591 (0.016)	0.483 (0.058)	-0.461 (0.072)
Cystine	0.520 (0.039)	-0.690 (0.003)	0.678 (0.004)	-0.484 (0.057)	-0.594 (0.015)	0.467 (0.068)	-0.534 (0.033)
Phenylalanine	0.709 (0.002)	-0.618 (0.011)	0.722 (0.002)	-0.782 (<0.001)	-0.661 (0.005)	0.732 (0.001)	-0.530 (0.035)
Tyrosine	0.721 (0.002)	-0.825 (<0.001)	0.902 (<0.001)	-0.863 (<0.001)	-0.817 (<0.001)	0.778 (<0.001)	-0.720 (0.002)
Threonine	0.668 (0.005)	-0.623 (0.010)	0.796 (<0.001)	-0.759 (0.001)	-0.630 (0.009)	0.491 (0.053)	-0.526 (0.036)
Tryptophan	-0.324 (0.221)	0.593 (0.016)	-0.576 (0.019)	0.538 (0.031)	0.573 (0.020)	-0.508 (0.045)	0.570 (0.021)
Valine	0.797 (<0.001)	-0.665 (0.005)	0.748 (0.001)	-0.827 (<0.001)	-0.714 (0.002)	0.671 (0.004)	-0.574 (0.020)
Alanine	0.102 (0.706)	0.363 (0.167)	-0.125 (0.644)	0.214 (0.426)	0.373 (0.155)	-0.387 (0.139)	0.475 (0.063)
Arginine	-0.306 (0.249)	-0.024 (0.930)	-0.178 (0.509)	0.315 (0.234)	0.070 (0.796)	-0.100 (0.711)	-0.025 (0.926)
Aspartic acid	0.149 (0.582)	-0.420 (0.105)	0.503 (0.047)	-0.423 (0.103)	-0.395 (0.130)	0.209 (0437)	-0.431 (0.096)
Glutamic acid	-0.646 (0.007)	0.476 (0.063)	-0.532 (0.032)	0.739 (0.001)	0.583 (0.018)	-0.664 (0.005)	0.458 (0.074)
Glycine	-0.584 (0.017)	0.813 (<0.001)	-0.789 (<0.001)	0.777 (<0.001)	0.803 (<0.001)	-0.830 (<0.001)	0.745 (0.001)
Proline	0.287 (0.281)	-0.389 (0.137)	0.148 (0.583)	-0.470 (0.066)	-0.511 (0.043)	0.053 (0.846)	-0.548 (0.028)
Serine	0.378 (0.148)	-0.585 (0.017)	0.723 (0.002)	-0.560 (0.024)	-0.527 (0.036)	0.404 (0.121)	-0.501 (0.048)
Crude fat	0.812 (<0.001)	-0.866 (<0.001)	0.940 (<0.001)	-0.903 (<0.001)	-0.858 (<0.001)	0.847 (<0.001)	-0.734 (0.001)
12:00	-0.230 (0.391)	0.761 (0.001)	-0.575 (0.020)	0.478 (0.061)	0.689 (0.003)	-0.442 (0.087)	0.758 (0.001)
14:00	-0.382 (0.144)	0.619 (0.011)	-0.528 (0.036)	0.667 (0.005)	0.675 (0.004)	-0.520 (0.039)	0.680 (0.004)
15:00	-0.769 (<0.001)	0.911 (<0.001)	-0.901 (<0.001)	0.897 (<0.001)	0.908 (<0.001)	-0.804 (<0.001)	0.817 (<0.001)

	LMWSDF	S-NCP	I-NCP	Cellulose	NSP	Klason lignin	DF
16:00	-0.777 (<0.001)	0.870 (<0.001)	-0.850 (<0.001)	0.926 (<0.001)	0.905 (<0.001)	-0.749 (0.001)	0.815 (<0.001)
17:00	-0.853 (<0.001)	0.801 (<0.001)	-0.849 (<0.001)	0.868 (<0.001)	0.816 (<0.001)	-0.912 (<0.001)	0.661 (0.005)
18:00	-0.638 (0.008)	0.721 (0.002)	-0.862 (<0.001)	0.750 (0.001)	0.686 (0.003)	-0.803 (<0.001)	0.580 (0.019)
20:00	0.642 (0.007)	-0.650 (0.006)	0.657 (0.006)	-0.646 (0.007)	-0.645 (0.007)	0.465 (0.070)	-0.557 (0.025)
22:00	0.701 (0.002)	-0.866 (<0.001)	0.879 (<0.001)	-0.799 (<0.001)	-0.829 (<0.001)	0.710 (0.002)	-0.748 (0.001)
24:00	-0.851 (<0.001)	0.799 (<0.001)	-0.820 (<0.001)	0.875 (<0.001)	0.828 (<0.001)	-0.920 (<0.001)	0.677 (0.004)
16:01	0.648 (0.007)	-0.873 (<0.001)	0.851 (<0.001)	-0.802 (<0.001)	-0.846 (<0.001)	0.725 (0.001)	-0.787 (<0.001)
18:1n9	0.814 (<0.001)	-0.921 (<0.001)	0.867 (<0.001)	-0.918 (<0.001)	-0.938 (<0.001)	0.795 (<0.001)	-0.841 (<0.001)
20:1n9	0.750 (0.001)	-0.874 (<0.001)	0.901 (<0.001)	-0.910 (<0.001)	-0.882 (<0.001)	0.866 (<0.001)	-0.784 (<0.001)
22:1n9	0.732 (0.001)	-0.879 (<0.001)	0.885 (<0.001)	-0.873 (<0.001)	-0.874 (<0.001)	0.889 (<0.001)	-0.779 (<0.001)
24:1n	0.279 (0.296)	-0.591 (0.016)	0.552 (0.027)	-0.499 (0.049)	-0.560 (0.024)	0.534 (0.033)	-0.569 (0.022)
18:2n6	-0.784 (<0.001)	0.920 (<0.001)	-0.858 (<0.001)	0.890 (<0.001)	0.927 (<0.001)	-0.786 (<0.001)	0.838 (<0.001)
18:3n3	0.615 (0.011)	-0.778 (<0.001)	0.841 (<0.001)	-0.730 (0.001)	-0.733 (0.001)	0.769 (0.001)	-0.650 (0.006)
20:2n6	0.326 (0.217)	-0.326 (0.217)	0.307 (0.247)	-0.359 (0.172)	-0.349 (0.186)	0.277 (0.299)	-0.306 (0.249)
20:3n6	-0.887 (<0.001)	0.760 (0.001)	-0.846 (<0.001)	0.868 (<0.001)	0.781 (<0.001)	-0.903 (<0.001)	0.606 (0.013)
Fructose	-0.823 (<0.001)	0.694 (0.003)	-0.739 (0.001)	0.775 (<0.001)	0.717 (0.002)	-0.771 (<0.001)	0.558 (0.025)
Glucose	-0.102 (0.706)	0.153 (0.571)	-0.198 (0.461)	-0.155 (0.566)	-0.012 (0.965)	-0.088 (0.747)	-0.060 (0.826)
Sucrose	0.293 (0.271)	-0.414 (0.111)	0.415 (0.110)	-0.615 (0.011)	-0.512 (0.043)	0.162 (0.550)	-0.536 (0.032)

*Red: correlation coefficient > |0.900|; orange: correlation coefficient = |0.700–0.800|; yellow: correlation coefficient = |0.500-0.700|.
